# Text Sentiment Analysis Based on a New Hybrid Network Model

**DOI:** 10.1155/2022/6774320

**Published:** 2022-12-28

**Authors:** Yancong Zhou, Qian Zhang, Dongdong Wang, Xiaoying Gu

**Affiliations:** ^1^School of Information Engineering, Tianjin University of Commerce, Tianjin 300134, China; ^2^Information Center, Hebei Provincial Tax Service State Taxation Administration, Shijiazhuang 050046, China

## Abstract

The research of text sentiment analysis based on deep learning is increasingly rich, but the current models still have different degrees of deviation in understanding of semantic information. In order to reduce the loss of semantic information and improve the prediction accuracy as much as possible, the paper creatively combines the doc2vec model with the deep learning model and attention mechanism and proposes a new hybrid sentiment analysis model based on the doc2vec + CNN + BiLSTM + Attention. The new hybrid model effectively exploits the structural features of each part. In the model, the understanding of the overall semantic information of the sentence is enhanced through the paragraph vector pretrained by the doc2vec structure which can effectively reduce the loss of semantic information. The local features of the text are extracted through the CNN structure. The context information interaction is completed through the bidirectional cycle structure of the BiLSTM. The performance is improved by allocating weight and resources to the text information of different importance through the attention mechanism. The new model was built based on Keras framework, and performance comparison experiments and analysis were performed on the IMDB dataset and the DailyDialog dataset. The results have shown that the accuracy of the new model on the two datasets is 91.3% and 93.3%, respectively, and the loss rate is 22.1% and 19.9%, respectively. The accuracy on the IMDB datasets is 1.0% and 0.5% higher than that of the CNN-BiLSTM-Attention model and ATT-MCNN-BGRUM model in the references. Comprehensive comparison has shown the overall performance is improved, and the new model is effective.

## 1. Introduction

Sentiment analysis, also known as sentiment polarity analysis, predicts the sentiment polarity of text by acquiring, processing, analyzing, summarizing, reasoning, and so on. In real life, based on the results of sentiment analysis, leaders can make more targeted decisions. In recent years, with the continuous development of artificial intelligence, sentiment analysis has gradually entered the practical application. It is based on the industry data analysis and the key technologies such as natural language processing and in-depth learning. By classifying the emotional attitudes of different data, the intelligent service system based on sentiment analysis can provide decision-making basis for decision-makers and provide more human-oriented services for ordinary users. Thus, to improve service efficiency and service quality as a whole [1], the core of sentiment analysis is sentiment classification. The difficulty is how to understand the emotional information of different words in the text [2]. At present, from the perspective of techniques, there are three kinds of text sentiment analysis methods, namely, sentiment analysis method based on emotional dictionary, traditional machine learning, and deep learning, respectively.

For the sentiment analysis method based on emotional dictionary, Yang and Zhang introduced SentiWordNet emotional dictionary to build a model and analyzed the emotional words and sentences according to it [3]. The main point of this method is to build a dictionary containing words and corresponding emotional information, and the quality of the dictionary directly determines the effect of sentiment classification. Because there is always a bias between the emotional dictionary and the actual application, the result of sentiment classification based on the method is often poor. On the sentiment analysis method based on traditional machine learning, Sudhir and Suresh have compared the models on IMDB (Internet Movie Database) data set [4]. On the basis of considering special words, such as affective words, negative words, and degree adverbs, this method also makes a comprehensive evaluation of most other words in the text, which is suitable for the processing of long text [5]. This method requires manual selection, labeling, and extraction of emotional features, and the results of the final emotional classification are relatively general.

In the sentiment analysis methods based on deep learning, the key is to establish deep learning models and complete the text sentiment classification through model training. This method is based on a deep neural network to simulate the network structure of the human brain and automatically mines the deep-seated and relevant semantic features of text information. So, the results of emotional classification are often better than that of sentiment analysis methods based on emotional dictionary and traditional machine learning [6]. With the deepening of the deep learning research, sentiment analysis based on deep learning models has gradually developed [7]. Kim and Jeong proposed sentiment analysis based on the CNN (Convolutional Neural Network) model [8]. Alireza et al. proposed a weighted convolutional neural network for multimodal sentiment analysis based on integrated transfer learning [9]. It has been proved that the CNN model can effectively understand the local features of text information for sentiment classification, but it is not close enough to the context information, and the accuracy of sentiment classification is not high. Khan et al. used CNN + LSTM architecture to conduct in-depth sentiment analysis of information shared on social media. They proved that the LSTM (Long Short-Term Memory) model could effectively interact with context information and the hybrid model had better effect than a single neural network model [10]. Gao et al. conducted aspect layer sentiment analysis to the short text based on CNN + BiGRU and proved that the two-way information interaction mechanism could effectively improve the results of emotional classification, but the information processing on the input layer is too single [11]. Kalaiarasu et al. extracted features through word2vec (Word Embeddings), using the improved new convolution neural network for sentiment analysis. They proved that the pretrained word vector could better quantitatively describe the relationship between different words, improve the input layer structure, and greatly improve the results of emotional classification, but there was a certain loss of text information [12]. Wang and Liu proposed a method based on doc2vec (Paragraph Vector) and the deep neural network to analyze the emotion of text information, which not only reduced the training cost but also had high efficiency. It proved that the doc2vec model could well reduce the loss of information [13], but the accuracy of sentiment classification is low. Fan proposed a sentiment analysis method based on the BERT + BLSTM + Attention model and introduced attention mechanism. They proved that different texts were given different weights to extract the relatively important parts of the input text, which could improve the accuracy of sentiment classification [14]. However, the model which can effectively integrate the advantages of each model and not only improve the accuracy but also effectively reduce the semantic loss and increase the expression ability of text features has not been seen so far.

The main purpose of this paper is to reduce the loss of semantic information as much as possible on the premise of improving the accuracy of emotion classification. This study focuses on the establishment and training process of the hybrid deep learning model which introduces the doc2vec model and attention mechanism. The input layer structure of the general deep learning model is improved through the doc2vec paragraph vector model to reduce the loss of semantic information. The CNN model is used to enhance the local feature expression ability of text information, and the BiLSTM model is added to make up for the lack of close connection of context information in the CNN model. By adding attention mechanism, text information of different importance is reasonably allocated. Finally, the new model is compared with other existing models on two public datasets, which then analyze the results of different models to verify the effectiveness of the proposed model.

The main contributions of this work are summarized as follows:

1. It innovatively integrates the doc2vec model with the deep learning model and attention mechanism to increase the ability of text feature expression while minimizing the loss of semantic information.
2. performs verification experiments on the IMDB and the DailyDialog public data sets. The results show that the doc2vec + CNN + BiLSTM + Attention model can effectively complete the task of sentiment analysis.
3. Itcompares the new model with different sentiment analysis models, and the results prove that the doc2vec + CNN + BiLSTM + Attention model is more stable and improves the accuracy of text emotion classification.

## 2. A Hybrid Deep Learning Model Introduced Doc2vec Model and Attention Mechanism

**Figure 1:**
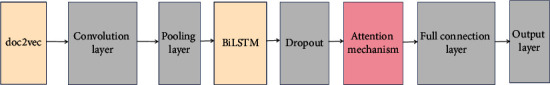
Structure of deep learning hybrid model with doc2vec model and attention mechanism.

Through the research on the sentiment analysis at home and abroad, an innovative hybrid deep learning model combined with the doc2vec model and attention mechanism is proposed. The model integrates the characteristics of each model to improve the accuracy of sentiment classification. The structure of the model is shown in Figure 1.

The model is mainly composed of doc2vec model, CNN model, BiLSTM (Bidirectional Long Short-Term Memory) model, and attention mechanism. First, text information is transformed into text vector matrix through the doc2vec model, which is used as embedded layer. Secondly, through the CNN model, feature extraction is carried out in the convolution layer, information filtering is carried out in the pooling layer, and the feature dimension is reduced. Then, the context information is exchanged through the cell gating mechanism in the BiLSTM model, and the dropout technology is used to prevent over fitting. Finally, the attention mechanism is used to allocate resources for text information of different importance, then the full connection layer is added, and the emotional classification results are output through the classifier.

### 2.1. Model Representation

For the dataset *D* with given text information, it contains *m* text sentences denoted as *X*{*x*_1_, *x*_2_,…, *x*_*m*_} and the corresponding emotional tags of each text sentence denoted as *Y*{*y*_1_, *y*_2_,…, *y*_*m*_}, in which each text sentence *x*_*i*_ is composed of *n* words denoted as {*x*_*i*1_, *x*_*i*2_,…, *x*_*in*_}, and the final objective function is shown in the following formula :

(1) $$\begin{array}{c} P\left(Y\left|X\right.\right)=\arg\max_{\theta }f\left(Y\left|X\right.;\theta \right)\text{,} \end{array}$$

where *θ* represents all parameters involved in the model and *f*(*∗*) is the mathematical expression of the network model.

### 2.2. Doc2vec Model

The doc2vec paragraph vector model [15] is introduced into the hybrid model to further improve the semantic understanding of datasets. Compared with the word2vec word vector model, the doc2vec paragraph vector model has a simpler training method. It accepts sentences of different lengths as training samples and trains the corresponding feature vectors directly from the corpus, with less loss of information.

**Figure 2:**
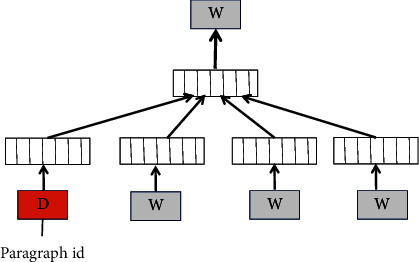
PV-DM model structure.

Doc2vec is proposed based on word2vec, and there are two training methods. One is the PV-DM (Distributed Memory Model of Paragraph Vectors) model, which predicts the probability of words given paragraph vectors and context words, similar to the CBOW (Continuous Bag-of-Words Model) model in word2vec. The specific structure is shown in Figure 2.

**Figure 3:**
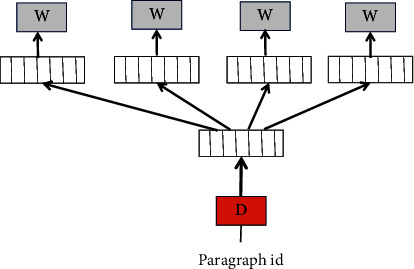
PV-DBOW model structure.

The other is the PV-DBOW (Distributed Bag of Words of Paragraph Vector) model, which predicts the probability of a group of random words in a paragraph given a paragraph vector, similar to the Skip-gram (Continuous Skip-gram Model) model in word2vec. The specific structure is shown in Figure 3.

This paper selects the PV-DM method in the doc2vec paragraph vector model for training. The preprocessed data set is directly used as the training corpus. Through the existing doc2vec model in the Gensim database, the training corpus is saved in the sentences using the TaggedLineDocument function. Then, the minimum word frequency, maximum distance, feature vector dimension, accelerated training method, and other parameters are set. In addition, the parameters of training model are all default values. Thus, the text vector space model is generated. Finally, the doc2vec paragraph vector model after training is saved. The specific training process is as follows:

First, given the initial words for training, assuming that there are *M* known words, the ultimate goal is to maximize the average logarithmic probability of the known words to predict the target words. The specific calculation is shown in the following formula :

(2) $$\begin{array}{c} \frac{1}{M}\overset{M-k}{\underset{m=k}{\sum }}\log\;P\left(\omega_{m}\left|\omega_{m-k}\right.,\cdot \cdot \cdot ,\omega_{m+k}\right)\text{.} \end{array}$$

Secondly, for each output word *ω*_*i*_, the average value of the word vector is extracted from the word vector matrix *W* by function *f*. In addition, *a* and *b* represent parameters, and the specific calculation is shown in the following formula :

(3) $$\begin{array}{c} y=a+bf\left(\omega_{m-k},\cdot \cdot \cdot ,\omega_{m+k};W\right)\text{.} \end{array}$$

Next, the word *ω*_*m*_ is predicted by the softmax function, and *y*_*ω*_*i*__ represents the nonstandardized logarithmic function. The specific calculation is as shown in the following formula :

(4) $$\begin{array}{c} P\left(\omega_{m}\left|\omega_{m-k}\right.,\cdot \cdot \cdot ,\omega_{m+k}\right)=\frac{e^{y_{\omega_{m}}}}{\sum_{i}e^{y_{\omega_{i}}}}\text{.} \end{array}$$

Finally, the paragraph vector paragraph id is added. This paragraph vector not only has a fixed length but also has the same length as the word vector, so it has better adaptability to new data. In formula (3), function *f* is jointly constructed by paragraph matrix *V* and word matrix *W*.

**Figure 4:**
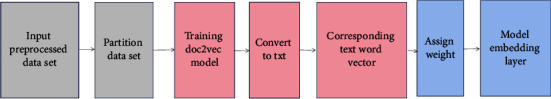
Process diagram of doc2vec layer.

To sum up, the doc2vec paragraph vector model is selected. First, the data set preprocessed is divided through the train_test_split function in Python's sklearn library. Secondly, the trained doc2vec paragraph vector model is converted into a visualized txt document, and the word vector of each text is mapped to the trained model one by one through the Tokenizer word splitter. Finally, according to the matching results, weight is allocated. Then, the embedded layer of the model for subsequent training is received. The specific process is shown in Figure 4.

### 2.3. CNN Model

**Figure 5:**
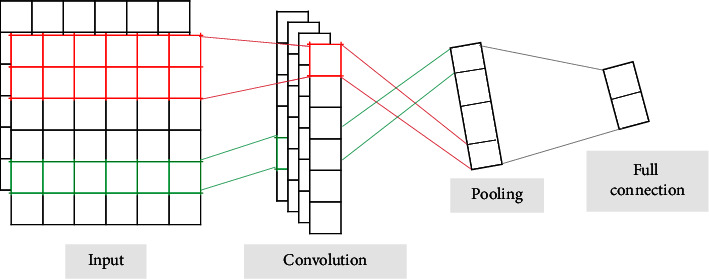
CNN model structure.

The local features of text information are extracted through the CNN model. The CNN is a feed forward neural network that performs translation classification on input information [16]. The specific structure is shown in Figure 5, which mainly includes the following four parts:

(1) Input Layer: This layer takes the output of doc2vec embedded layer as the input of the CNN layer. The word vector of each comment in the dataset is denoted as *x*_*i*_, *x*_*i*_ ∈ *R*^*n*×*d*^, where *n* is the number of words and *d* is the vector dimension.
(2) Convolution Layer: This layer selects the Convolution1D function of the Keras Library in Python and extracts the features of the input layer data through the filter. The calculation function is shown in the following formula :
  (5) $$\begin{array}{c} J_{i}=f\left(\omega \times x_{i:i+g-1}+b\right)\text{,} \end{array}$$
where *ω* represents the convolution kernel, *g* represents the size of the convolution kernel, *x*_*i*:*i*+*g*−1_ is the sentence vector composed of *i* to *i*+*g* − 1 words, *b* represents the offset term, and the obtained feature matrix *J* represents {*c*_1_, *c*_2_,…, *c*_*n*−*g*+1_}.
Relu [17] is selected as the activation function, which is simple and can effectively alleviate the disappearance of gradient. The calculation function is as shown in the following formula :
  (6) $$\begin{array}{c} ReLU\left(x\right)=\left\{\begin{array}{cc} x\text{,} & if\geq 0\text{,}\\ 0\text{,} & if<0\text{.} \end{array}\right. \end{array}$$
(3) Pooling Layer: This layer selects the MaxPooling1D pooling function of the Keras Library in Python, selects and filters the features extracted from the convolution layer, and reduces the feature dimension. The calculation function is as follows:
  (7) $$\begin{array}{c} M=\max\;\left(c_{1},c_{2},\cdots ,c_{n-g+1}\right)=\max\left\{J\right\}\text{.} \end{array}$$
(4) Full Connection Layer: This layer connects the *M*_*i*_ vector after the previous layer into vector *Q*, which is used as the input information of the BiLSTM layer. The calculation function is shown in the following formula :
  (8) $$\begin{array}{c} Q=\left\{M_{1},M_{2},\cdots ,M_{n}\right\}\text{.} \end{array}$$

### 2.4. BiLSTM Model

**Figure 6:**
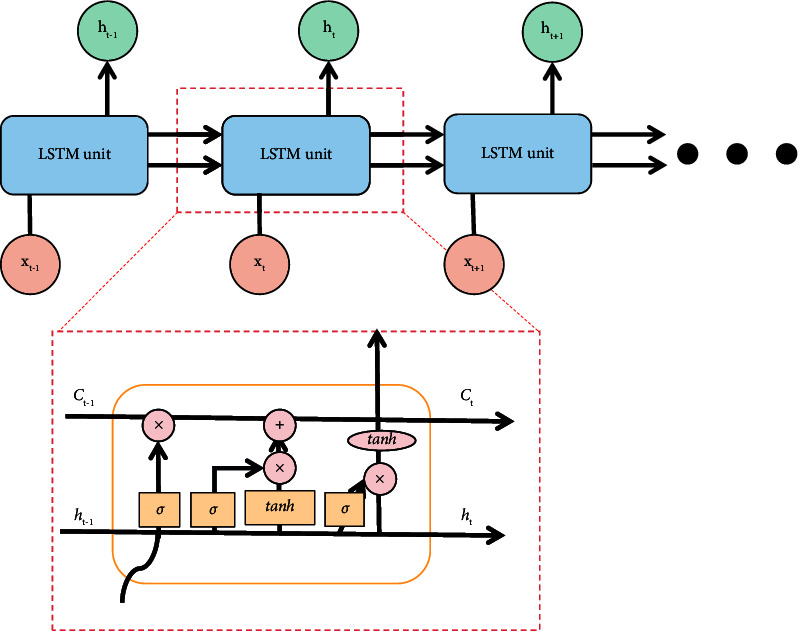
LSTM model structure.

LSTM is derived from RNN (Recurrent Neural Network), which is a time-based recurrent neural network that can solve long-term dependence. Compared with the original RNN, LSTM adds a cell state and realizes information interaction through the gating mechanism. The model structure is shown in Figure 6, which is composed of forgetting gate, input gate, and output gate.

**Figure 7:**
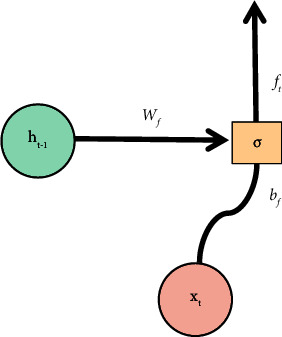
LSTM model-forgetting gate.

**Figure 8:**
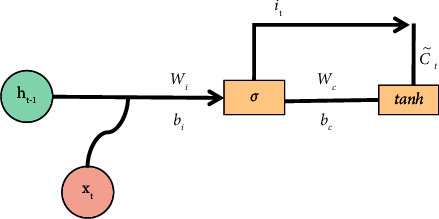
LSTM model-input gate 1.

**Figure 9:**
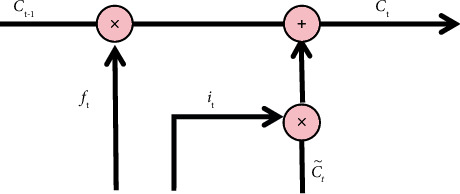
LSTM model-input gate 2.

(1) Forgetting Gate: It discards useless information mentioned above through this step.
The structure of the forgetting gate is shown in Figure 7. The gate reads *h*_*t*−1_ and *x*_*t*_ first, and then output *f*_*t*_ through *σ*, and the result value is between 0 and 1. Here, *h*_*t*−1_ represents the output information of the last cell state, *x*_*t*_ represents the input information of the current cell state, *σ* represents sigmoid function, the result value 0 indicates complete rejection, and the result value 1 indicates complete retention. The forgetting gate function *f*_*t*_ is expressed as formula (9), where *W*_*f*_ represents the weight and *b*_*f*_ represents the offset.
  (9) $$\begin{array}{c} f_{t}=\sigma \left(W_{f}\cdot \left[h_{t}-1,x_{t}\right]+b_{f}\right)\text{.} \end{array}$$
(2) Input Gate: This step completes the update of cell status information, which is divided into the following two operations:
  (a) As shown in Figure 8, we determine the updated information. First, we get *i*_*t*_ by *σ* layer. The result value is between 0 and 1, which is used to determine what information to be updated. Then, a vector $\tilde{c_{t}}$ is generated by tanh function to filter the input candidate information. Here, *σ* indicates sigmoid function, result value 0 indicates unimportant information, and result value 1 indicates important information. The input gate function is denoted as formula (10), and the input candidate information function $\tilde{c_{t}}$ is denoted as formula (11). Here, *W*_*i*_ and *W*_*c*_ represent weights and *b*_*i*_ and *b*_*c*_ represent offsets.
    (10) $$\begin{array}{c} i_{t}=\sigma \left(W_{i}\cdot \left[h_{t-1},x_{t}\right]+b_{i}\right)\text{,} \end{array}$$
    (11) $$\begin{array}{c} \tilde{C_{t}}=\tanh\left(W_{c}\cdot \left[h_{t-1},x_{t}\right]+b_{c}\right)\text{.} \end{array}$$
  (b) As shown in Figure 9, we update the cell status. First, we multiply the old cell state *C*_*t*−1_ by the forgetting gate function *f*_*t*_ and discard the useless above information. Then, we multiply the input gate function by the input candidate information function $\tilde{c_{t}}$ to determine the updated information. Finally, we combine the two parts to realize the transformation from *C*_*t*−1_ to *C*_*t*_ and complete the update of cell state. The function is as shown in the following formula :
    (12) $$\begin{array}{c} C_{t}=f_{t}*C_{t-1}+i_{t}*{\hat{C}}_{t}\text{.} \end{array}$$
(3) Output Gate: It determines the output information through this step.

**Figure 10:**
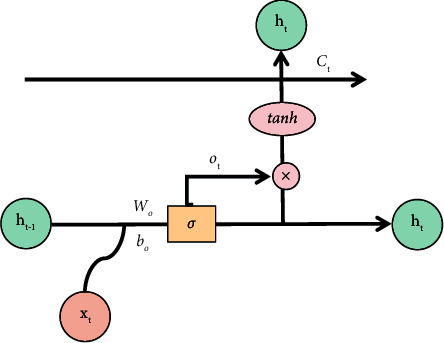
LSTM model-output gate.

The structure of the output gate is shown in Figure 10. First, we get *o*_*t*_ through the *σ* layer sigmoid function, determining which part of the cell state to be output. Then, we process the cell state *C*_*t*_ at time *t* by tanh function and obtain a value between −1 and 1. Finally, these two parts are multiplied to obtain *h*_*t*_, which is used to determine the final output information of cell state. The output gate function *o*_*t*_ is denoted as formula (13), and the output information function *h*_*t*_ is denoted as formula (14), where *W*_*o*_ represents weight and *b*_*o*_ represents offset.

(13) $$\begin{array}{c} o_{t}=\sigma \left(W_{o}\cdot \left[h_{t-1},x_{t}\right]+b_{o}\right)\text{,} \end{array}$$

(14) $$\begin{array}{c} h_{t}=o_{t}*\;\tanh\left(C_{t}\right)\text{.} \end{array}$$

**Figure 11:**
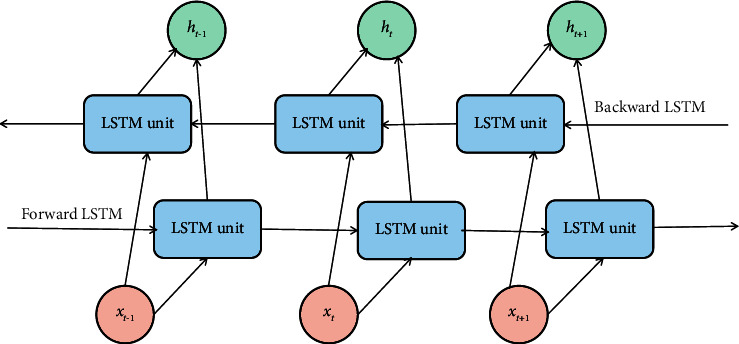
BiLSTM model structure.

Compared with the unidirectional LSTM structure, the BiLSTM structure can obtain the long-term information of the text in both directions and reduce the error as much as possible. The BiLSTM model structure is shown in Figure 11.

Each cycle of BiLSTM is composed of two parts, namely, front hidden state ${\overset{⟶}{h}}_{t}$ and rear hidden state ${\overset{⃖}{h}}_{t}$, and both of them are LSTM network structures [18]. Similar to the unidirectional LSTM, ${\overset{⟶}{h}}_{t}$ means to update the cell status and information from the front to the back along the direction of time *t* from time 0, while ${\overset{⃖}{h}}_{t}$ means to update the cell status and information from the back to the front along the direction of time 0 from time *t*. Finally, the splicing results of time *t*${\overset{⟶}{h}}_{t}$ and ${\overset{⃖}{h}}_{t}$ are output. The calculation function is as follows:

(15) $$\begin{array}{c} h_{t}={\overset{⟶}{h}}_{t}⊕{\overset{⃖}{h}}_{t}\text{.} \end{array}$$

To sum up, the BiLSTM layer is selected. That is, the bidirectional function of the Keras library in Python is added in front of the unidirectional LSTM layer. The local semantic information extracted from the CNN layer is processed through a two-way loop to obtain the context semantic information of long text and measures of preventing overfitting are added.

### 2.5. Attention Mechanism

Attention mechanism is a data analysis method of deep learning. First, we focus on important local information and then combine the information from different regions to form an overall evaluation [19].

**Figure 12:**
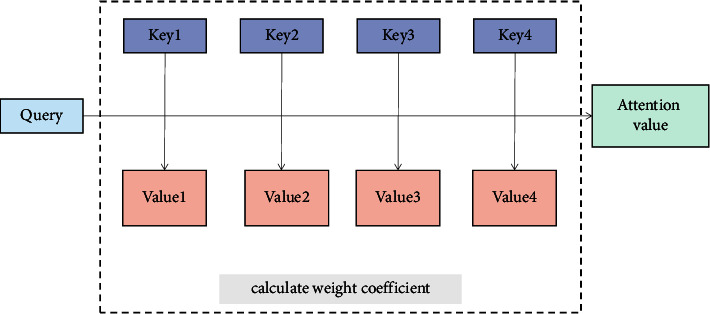
Computational process of attention mechanism.

We take the output information of the previous layer as the input information of this layer and assume that the input information is in the form of (Key, Value), where Key represents keyword, Value represents weight, and set vector Query represents the query. Then, the specific calculation process of attention level is shown in Figure 12.

First, we calculate the similarity between each vector Query and the Key of each input information through function *F* and get the attention score *e*_*ti*_, as shown in the following formula :

(16) $$\begin{array}{c} e_{ti}=F\left(q_{t},k_{i}\right)\text{.} \end{array}$$

Secondly, we normalize the attention score *e*_*ti*_ by the softmax function to obtain the weight coefficient *α*_*ti*_ of the Value of the input information. The larger the weight coefficient, the higher attention should be paid to this part of the information, as shown in the following formula :

(17) $$\begin{array}{c} \alpha_{ti}=soft\;\max\left(e_{ti}\right)=\frac{\exp\left(e_{ti}\right)}{\overset{n}{\underset{i=1}{\sum }}\exp\left(e_{ti}\right)}\text{.} \end{array}$$

Finally, the Value of each input information is weighted and summed with its corresponding weight coefficient to obtain the attention value, as shown in the following formula :

(18) $$\begin{array}{c} A\left({\left(\text{Key},\text{Value}\right),q}_{t}\right)=\overset{N}{\underset{i=1}{\sum }}\alpha_{ti}v_{i}\text{.} \end{array}$$

To sum up, the attention mechanism uses the attention function of the Keras library in Python to allocate the weight and calculate the attention value of the features obtained by the BiLSTM layer. It then judges the importance of the text information according to the size of the results to complete the corresponding resource allocation. Finally, we obtain the result of text sentiment classification through the output layer.

## 3. Experiments and Analysis

In this paper, the deep hybrid model, which integrates doc2vec model, deep learning model, and attention mechanism, is applied to sentiment analysis tasks. Different models are compared on the same dataset to verify their effectiveness. The hybrid model runs on windows10 system and is built in Python language based on the deep learning framework Keras in PyCharm integrated development environment.

### 3.1. Experimental Data

The experimental datasets are the IMDB movie review dataset and the DailyDialog multiround dialog text dataset. The IMDB dataset contains 50000 movie emotional reviews, and emotional polarity is a secondary category. Among them, about 25000 emotional comments are negative polarity, marked as 0, and the remaining 25000 emotional comments are positive polarity, marked as 1. The DailyDialog dataset contains tens of thousands of dialogues, and each dialogue is divided into several sentences, each of which corresponds to seven emotional attitudes. Among them, we delete the sentences marked with 0 and classify the sentences marked with 1∼3 as negative polarity and 4∼6 as positive polarity.

Usually, the original dataset needs to be preprocessed before the formal sentiment analysis experiment. The preprocessing process of the experimental dataset mainly includes three parts, namely, corpus cleaning, text segmentation, and removal of pause words, as follows:

1. Corpus Cleaning: We delete the html tags and non-English characters (punctuation, numbers, etc.) in the dataset and convert all English characters into lowercase mode. The dataset after corpus cleaning can eliminate the interference of irrelevant texts and improve the accuracy of sentiment classification of the model.
2. Text Segmentation: Each comment in the English dataset is directly segmented according to the space. For example, “what a waste of precious time” text segmentation results in [“what,”“a,”“waste,” “of,”“precious,”“time”]. Text word segmentation is the core content of data set preprocessing. The result determines the difference of semantic understanding and directly affects the accuracy of sentiment classification of the model.
3. Remove Stop Words: We use the English stop words list in NLTK to delete words that have no actual impact on semantic understanding in the dataset. For example, “this,” “was,” and “a” in “this was a discarding film” belong to stop words that need to be deleted. The final result is “discarding film.” The removal of stop words can reduce the space occupied by the dataset and improve the efficiency of model sentiment analysis.

The IMDB dataset and DailyDialog dataset are divided into training set, test set, and verification set according to 8 : 1 : 1, as shown in Table 1.

### 3.2. Experimental Parameter Setting

In this paper, the model's training parameters are set as following, including the doc2vec + CNN + BiLSTM + Attention model training parameters (see Table 2) and the doc2vec model training parameters (see Table 3).

### 3.3. Evaluation Index

#### 3.3.1. Accuracy

Accuracy is the most commonly used indicator to measure the performance of a model, which indicates the proportion of correctly predicted data in all data. Generally speaking, the higher the accuracy is, the better the model effect is. The calculation method is shown in the following formula :

(19) $$\begin{array}{c} Acc=\frac{TP+TN}{TP+FP+FN+TN}\text{,} \end{array}$$

where TP refers to the number of samples whose actual emotional polarity is positive and predicted to be positive, that is, the number of samples of the real examples; FP represents the number of samples that are actually negative but are predicted to be positive, that is, the number of false positive examples; FN represents the number of samples that are actually positive but predicted to be negative, that is, the number of false counterexamples; TN represents the number of samples that are actually negative and predicted to be negative, that is, the number of true counterexamples [20]. The confusion matrix is shown in Table 4.

#### 3.3.2. Loss Rate

The loss rate describes the difference between the predicted value and the real value. The sentiment analysis problem studied in the paper belongs to the binary classification problem, so the binary cross entropy algorithm is selected as the loss function. The ultimate goal is to optimize all parameters, minimize the objective function (loss function) as far as possible, and guide the model to move towards convergence in the training process. The function is as the following formula :

(20) $$\begin{array}{c} Loss=-\frac{1}{N}\overset{N}{\underset{i=1}{\sum }}y_{i}\cdot \;\log\;\left(p\left(y_{i}\right)\right)+\left(1-y_{i}\right)\text{,} \end{array}$$

where *y* is the binary tag 0 or 1, and *p*(*y*) is the probability that the output belongs to the *y* tag.

### 3.4. Experimental Comparison Model

In this paper, different control groups are set up to conduct comparative experiments on the same data set to verify the performance of the new hybrid model based on doc2vec + CNN + BiLSTM + Attention. The control groups are divided into three groups, which are comparison experiments of sentiment analysis based on simple deep learning models, word2vec models, and doc2vec models, respectively.

#### 3.4.1. Comparative Experiments of Sentiment Analysis Based on Simple Deep Learning Models

**Figure 13:**
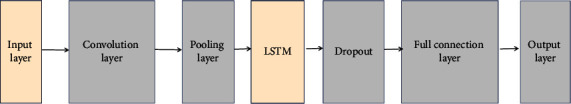
CNN + LSTM model structure.

In the experiments, CNN + LSTM hybrid model, CNN model, and LSTM model are selected for comparative experiments. The hybrid deep learning model is compared with the single deep learning models to verify whether the deep learning model can effectively combine the advantages of each part, and the effect is better than the single model. Among them, the structure of the CNN + LSTM model is shown in Figure 13.

In the experiment, firstly, the dataset is preprocessed and transformed into text vector matrix as the input layer of the model. Secondly, the convolution calculation is carried out by setting the corresponding parameters in the CNN layer, and the ReLU function is selected as the activation function, while the pool function is selected as the corresponding pool function in the pool layer to do the feature selection and dimension reduction. After obtaining the local semantic information of the text, the cell state is continuously updated through the gating mechanism of the LSTM layer, so as to obtain the semantic information of the long text. The dropout function is added to effectively prevent the over fitting in the model training process. Finally, the full connection layer sets units and activation functions, and the output layer obtains the emotional classification results.

#### 3.4.2. Comparative Experiments of Sentiment Analysis Based on word2vec Models

**Figure 14:**
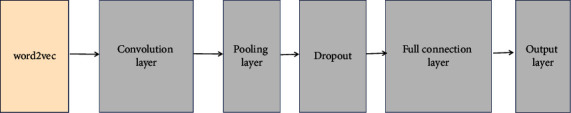
Word2vec + CNN model structure.

**Figure 15:**
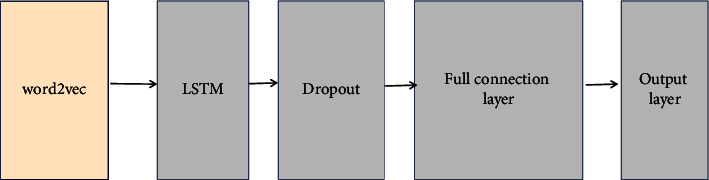
Word2vec + LSTM model structure.

**Figure 16:**
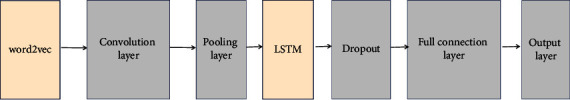
Word2vec + CNN + LSTM model structure.

The experiments combine the word2vec model with CNN model, LSTM model, and CNN + LSTM model, respectively, to verify the effectiveness of the word2vec word vector model. It can effectively avoid experimental contingency, reduce error, and make the results more objective, as follows:

1. Word2vec + CNN Model: Based on the CNN model, the trained word2vec word vector model is used as the embedding layer to replace the original input layer. The weight of the embedding layer is changed from the original randomly assigned vector to word2vec word vector. The convolution layer sets the corresponding parameters for convolution calculation, and the activation function selects the ReLU function. The pooling layer selects the corresponding pooling function for feature selection and dimension reduction and then adds the dropout layer. The full connection layer sets units and activation functions. Finally, the output layer obtains the sentiment classification results. The structure of the word2vec + CNN model is shown in Figure 14.
2. Word2vec + LSTM Model: Based on the LSTM model, the trained word2vec word vector model is used as the embedding layer to replace the original input layer. The weight of the embedding layer is not a randomly assigned vector but a word2vec word vector. We set LSTM layers and add dropout layer to prevent over fitting. The full connection layer sets units and activation functions, and finally the output layer obtains the sentiment classification results. The structure of the word2vec + LSTM model is shown in Figure 15.
3. Word2vec + CNN + LSTM Model: Based on the CNN + LSTM model, the trained word2vec word vector model is used as the embedding layer to replace the input layer of Figure 15, and the weight of the embedding layer is changed from the original randomly assigned vector to word2vec word vector. In addition, the model structure remains unchanged. The word2vec + CNN + LSTM model structure is shown in Figure 16.

#### 3.4.3. Comparative Experiments of Sentiment Analysis Based on doc2vec Models

**Figure 17:**
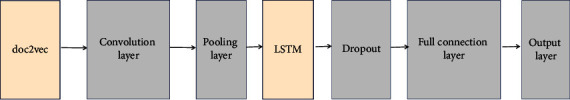
Doc2vec + CNN + LSTM model structure.

**Figure 18:**
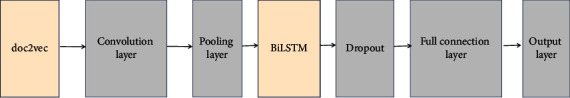
Doc2vec + CNN + BiLSTM model structure.

In the experiments, the doc2vec + CNN + LSTM model is compared with the word2vec + CNN + LSTM model to verify the effectiveness of the doc2vec paragraph vector model. Then, we set the doc2vec + CNN + BiLSTM model to verify the impact of bidirectional LSTM structure and attention mechanism on the performance of the model. The details are as follows:

1. Doc2vec + CNN + LSTM Model: Based on the word2vec + CNN + LSTM model, the trained doc2vec paragraph vector model is used as the embedding layer to replace the original word2vec word vector embedding layer. In addition, the model structure remains unchanged. The doc2vec + CNN + LSTM model structure is shown in Figure 17.
2. Doc2vec + CNN + BiLSTM Model: Based on the doc2vec + CNN + LSTM model, the original unidirectional LSTM layer is replaced by the BiLSTM layer. In addition, the model structure remains unchanged. The doc2vec + CNN + BiLSTM model structure is shown in Figure 18.

### 3.5. Analysis of Experimental Results

The model is evaluated by comparing the results of accuracy and loss rates of different models in the test set. The experimental results are shown in Table 5.

#### 3.5.1. Analysis of Experimental Results Based on Simple Deep Learning Models

**Figure 19:**
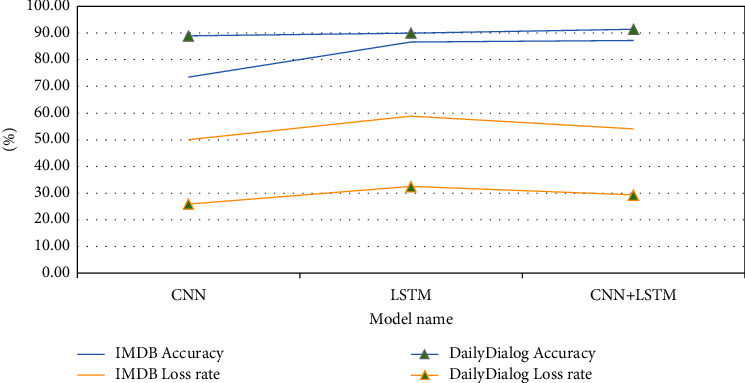
Experimental results based on simple deep learning models.

Experimental results based on simple deep learning models are as shown in Figure 19. According to Figure 19, on the IMDB dataset, the accuracy of a single LSTM model is 13.2% higher than that of a single CNN model, but the loss rate is 8.8% higher. The accuracy of the CNN + LSTM hybrid model is 13.7% higher than that of CNN model, 0.5% higher than that of the LSTM model, and the loss rate is 4% higher than that of the CNN model and 4.8% lower than that of the LSTM model.

On the DailyDialog dataset, the accuracy of a single LSTM model is 1.1% higher than that of a single CNN model, while the loss rate is 6.6% higher. The accuracy of the CNN + LSTM hybrid model is 2.5% higher than that of the CNN model, 1.4% higher than that of the LSTM model, and the loss rate is 3.4% higher than that of the CNN model and 3.2% lower than that of the LSTM model.

Compared with the local information of the text, the understanding of context information has a greater impact on the accuracy of sentiment classification. The CNN + LSTM hybrid model has the best performance [21]. It pays attention to both the local characteristics of information and the abovementioned time interval characteristics and then makes emotion prediction, which can effectively improve the performance of the model [22].

#### 3.5.2. Analysis of Experimental Results Based on word2vec Models

**Figure 20:**
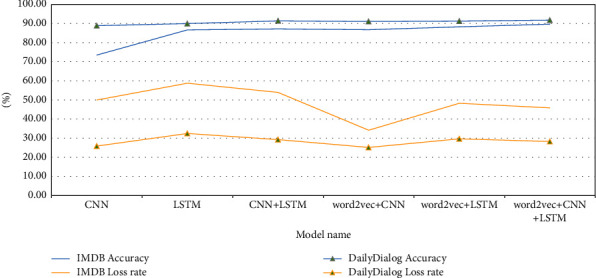
Experimental results based on word2vec models.

Experimental results based on word2vec models are as shown in Figure 20. According to Figure 20, on the IMDB dataset, the accuracy of the word2vec + CNN model is 13.3% higher than that of the separate CNN model, and the loss rate is 15.8% lower; compared with the LSTM model, the accuracy of the word2vec + LSTM model is 1.6% higher and the loss rate is 10.3% lower. The accuracy of the word2vec + CNN + LSTM model is 2.5% higher than that of the CNN + LSTM model, and the loss rate is 8.1% lower.

On the DailyDialog dataset, the accuracy of the word2vec + CNN model is 2.2% higher than that of the single CNN model, and the loss rate is 0.7% lower; compared with the LSTM model, the accuracy of word2vec + LSTM model is 1.3% higher and the loss rate is 2.8% lower. The accuracy of the word2vec + CNN + LSTM model is 0.4% higher than that of the CNN + LSTM model, and the loss rate is 1% lower.

It shows that the accuracy of the three models with word2vec pretraining word vector is significantly higher than that of the corresponding simple deep learning model. The word vector model pretrained by word2vec can better understand the semantic information and then effectively improve the structure of the simple deep learning input layer to get a higher performance model.

#### 3.5.3. Analysis of Experimental Results Based on doc2vec Models

In order to further explore the role of doc2vec model in reducing the loss of semantic information, we compare the doc2vec model with the word2vec model firstly.

**Figure 21:**
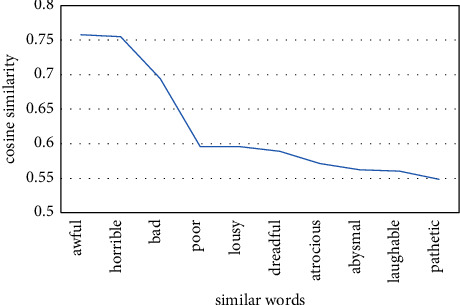
The top ten words with the highest similarity to terrible cosine in word2vec.

**Figure 22:**
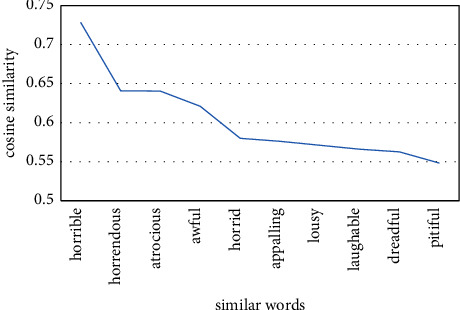
The top ten words with the highest similarity to terrible cosine in doc2vec.

On the IMDB dataset, we load the trained word2vec word vector model and the doc2vec paragraph vector model and get the cosine similarity between different words. For example, the top 10 words with the highest cosine similarity with “terrible” are shown in Figures 21 and 22.

For the same word “terrible,” the top ten words with the highest cosine similarity have changed. It indicates that there is a deviation in the understanding of semantic information between word2vec word vector model and doc2vec paragraph vector model. Word2vec pretrained word vectors can better understand the semantic information of the text than general word vectors and can quantitatively analyze the relationship between different words. However, word2vec ignores the influence of the order of words on semantic understanding, while doc2vec introduces paragraph vectors with variable sentence length, which is simpler and more flexible. It can improve the abovementioned problems and reduce the loss of semantic information. The deeper and more accurate the semantic understanding, the better the result of emotional classification of the model.

**Figure 23:**
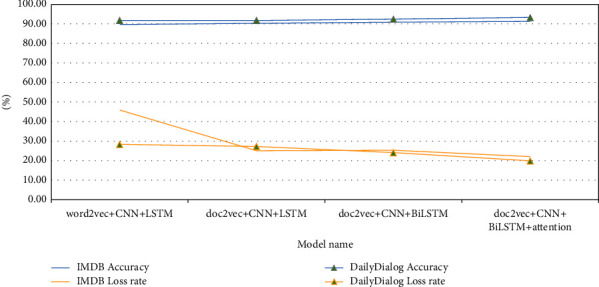
Experimental results based on doc2vec models.

Secondly, according to Figure 23, we compare and analyze the doc2vec + CNN + LSTM and the word2vec + CNN + LSTM model. On the IMDB dataset, the accuracy of the doc2vec + CNN + LSTM model is 0.6% higher than that of the word2vec + CNN + LSTM model, and the loss rate is 20.8% lower. On the DailyDialog dataset, the accuracy of the doc2vec + CNN + LSTM model is the same as that of the word2vec + CNN + LSTM model, and the loss rate is 1% lower. It shows that the gap between the predicted value and the real value of the doc2vec model is smaller, which can better retain the semantic information of the text, reduce the loss of information, and make the final model performance more stable.

Then, we compare and analyze the doc2vec + CNN + BiLSTM and the doc2vec + CNN + LSTM model. On the IMDB dataset, the accuracy of doc2vec + CNN + BiLSTM model is 0.6% higher than that of the doc2vec + CNN + LSTM model, but the loss rate is 0.2% higher. On the DailyDialog dataset, the accuracy of the doc2vec + CNN + BiLSTM model is 0.7% higher than that of doc2vec + CNN + LSTM model, and the loss rate is 3.2% lower. It shows that the bidirectional LSTM structure can better retain historical information, interact with context information, and improve the accuracy of sentiment classification than the unidirectional LSTM structure.

Finally, we compare and analyze the doc2vec + CNN + BiLSTM + Attention and the doc2vec + CNN + BiLSTM model. On the IMDB dataset, the accuracy of the doc2vec + CNN + BiLSTM + Attention model is 0.4% higher and the loss rate is 3.2% lower than that of the doc2vec + CNN + BiLSTM model. On the DailyDialog dataset, the accuracy of the doc2vec + CNN + BiLSTM + attention model is 0.8% higher than that of the doc2vec + CNN + BiLSTM model, and the loss rate is 4.2% lower. It shows that the introduced attention mechanism can better allocate weights and pay attention to important information, which greatly improves the overall performance of the model.

### 3.6. Comparative Analysis with Other Sentiment Analysis Models

The models based on doc2vec are compared with the models of some latest literature on the same IMDB dataset. The results of sentiment classification accuracy are analyzed to further illustrate the effectiveness of doc2vec + CNN + BiLSTM + attention hybrid model. The comparison literature models are as follows:

1. CNN-BiLSTM-Attention model in the document 《Text Sentiment Analysis Based on CNN-BiLSTM Network and Attention Model》 combines CNN, BiLSTM and attention mechanism [23].
2. ATT-MCNN-BGRUM model in the document 《Sentiment Analysis Based on Multi-channel Convolution and Bi-directional GRU with Attention Mechanism》 combines bidirectional GRU, multichannel convolution, and attention mechanism [24].

The comparison results of sentiment analysis models are shown in Table 6.

According to Table 6, the accuracy of sentiment classification of the abovementioned models is different due to different processing of input information, different neural network selection, or different internal structure design of the model, as follows:

1. Comparing the doc2vec + CNN + LSTM model with the CNN-BiLSTM-Attention model, we can see that the accuracy of the two models is consistent. It shows that the doc2vec paragraph vector model and attention mechanism have a great impact on the accuracy of sentiment classification.
2. Comparing the doc2vec + CNN + BiLSTM model with the CNN-BiLSTM-Attention model, it can be seen that the accuracy of doc2vec + CNN + BiLSTM model is 0.6% higher than that of the CNN-BiLSTM-Attention model. It shows that doc2vec pretrained paragraph vectors can better understand the relationship between words and sentences than general vectors, and significantly improve the accuracy of model classification.
3. Comparing the doc2vec + CNN + BiLSTM model with the ATT-MCNN-BGRUM model, it can be seen that the accuracy of doc2vec + CNN + BiLSTM model is 0.1% higher than that of the ATT-MCNN-BGRUM model. It shows that the structure and function of multichannel convolution and CNN, bidirectional GRU, and bidirectional LSTM are similar, but the doc2vec paragraph vector introduced can better understand semantic information and improve the performance of the model than the general attention mechanism.
4. Comparing the new hybrid model based on doc2vec + CNN + BiLSTM + attention in this paper with CNN-BiLSTM-Attention model and ATT-MCNN-BGRUM model in the literature, we can see that the accuracy of the new model is 1.0% and 0.5% higher than that of the literature models, respectively. It shows that the doc2vec paragraph vector model can effectively reduce the loss of semantic information, and the attention mechanism can reasonably allocate emotional weights to improve the final classification effect of the model.

In summary, the new doc2vec + CNN + BiLSTM + attention model can effectively play the structural features of each part and has good performance. The doc2vec structure enhances the understanding of the overall semantic information of the sentence through the paragraph vector. The CNN structure extracts the local features of the text. The BiLSTM structure completes the information interaction of the context through a two-way cycle. The attention mechanism allocates weights and resources according to the importance of the text information. In addition, the arrangement of the internal structure of the model is also more appropriate, thus greatly improves the performance of the model. Therefore, the doc2vec + CNN + BiLSTM + Attention model is a better model of text sentiment analysis with good performance.

## 4. Conclusion

This paper innovatively integrates the doc2vec model with the deep learning model and attention mechanism to increase the ability of text feature expression, minimize the loss of semantic information, and improve the accuracy of sentiment classification. The doc2vec + CNN + BiLSTM + attention hybrid model makes full use of the advantages of each structure. It correctly extracts the local features of the text information through CNN structure, enhances the interaction ability of the context information through the BiLSTM structure, better understands semantic information and reduces the semantic information loss through the doc2vec model, and reasonably allocates important resources through the attention mechanism. The accuracy is 91.3% and 93.3%, and the loss rate is 22.1% and 19.9% on the IMDB dataset and the the DailyDialog dataset, respectively. The accuracy of the hybrid model is 1.0% and 0.5% higher than that of the literature's models. The experimental results show that the new model effectively exerts the advantages of each part, improves the accuracy, and has stable performance. However, the new model mainly aims at the binary classification problem and has weak ability to deal with complex problems [25]. The processing speed is slow, and the prediction accuracy can be further improved. Therefore, it is necessary to further improve the algorithm in the future, such as expanding the binary classification problem to a multiclassification problem on the premise of ensuring the accuracy of sentiment classification, combining hardware to improve model speed [26], and combining domain knowledge [27] to further reduce semantic loss and improve accuracy.

## Figures and Tables

**Table 1:** Experimental data setting.

Dataset	Training set	Validation set	Testing set	Set total
IMDB	40000	5000	5000	50000
Dailydialog	13600	1700	1700	17000

**Table 2:** Training parameter settings of doc2vec + CNN + BiLSTM + Attention model.

Name	Parameter	Parameter value
doc2vec embedded layer	Embeddings_dim	200
	Max_len	200

Convolution layer	Filters	64
	Kernel_size	5
	Padding	Valid
	Activation	Relu
	Strides	1

Convolution layer	Pool_size	4

BiLSTM model	Units	128
	Dropout	0.5

Full connection layer	Units	64
	Activation	Relu

Output layer	Units	1
	Activation	Sigmoid

Training parameters	Optimizer	Adam
	Loss	Binary_cross-entropy
	Metrics	Accuracy
	Rate	0.01
	Batch_size	32
	Epoch	5

**Table 3:** Parameter settings of doc2vec model.

Name	Parameter value	Meaning
dm	1	Training algorithm
Min_count	1	Truncate dictionary
Window	10	The maximum distance between the current word and the prediction word in a sentence
Vector_size	300	Dimension of eigenvector
Sample	0.01	Configuration threshold of random downsampling of high frequency words
Negative	5	Set interference words
Workers	7	Parallel number of parameter control training

**Table 4:** Confusion matrix.

Physical truth	Forecast results	
	Positive emotional polarity	Negative emotional polarity
Positive emotional polarity	TP (real examples)	FN (false counterexamples)
Negative emotional polarity	FP (false positive samples)	TN (true counterexamples)

**Table 5:** Experimental results.

Model	IMDB		Dailydialog	
	Accuracy (%)	Loss rate (%)	Accuracy (%)	Loss rate (%)
CNN	73.5	50.0	88.9	25.9
LSTM	86.7	58.8	90.0	32.5
CNN + LSTM	87.2	54.0	91.4	29.3
Word2vec + CNN	86.8	34.2	91.1	25.2
Word2vec + LSTM	88.3	48.3	91.3	29.7
Word2vec + CNN + LSTM	89.7	45.9	91.8	28.3
Doc2vec + CNN + LSTM	90.3	25.1	91.8	27.3
Doc2vec + CNN + BiLSTM	90.9	25.3	92.5	24.1
Doc2vec + CNN + BiLSTM + Attention	**91.3**	**22.1**	**93.3**	**19.9**

The bold values mean they are the best results.

**Table 6:** Comparison results of sentiment analysis models.

Model	Accuracy (%)
CNN-BiLSTM-Attention [23]	90.3
Doc2vec + CNN + LSTM	**90.3**
ATT-MCNN-BGRUM [24]	90.8
Doc2vec + CNN + BiLSTM	**90.9**
Doc2vec + CNN + BiLSTM + Attention	**91.3**

## Data Availability

The data involved in the research are public data. They do not involve ethics and commercial secrets. The IMDB dataset can be downloaded through the URL: http://movie.douban.com/doulist/1518184/. The DailyDialog dataset can be downloaded through the URL: https://github.com/PaddlePaddle/Research/tree/master/NLP/Dialogue-PLATO

## References

[1] Zhang H., Ding Y., Zhang Y., Shi F. (2021). Design and implementation of E-commerce intelligent customer service system based on deep neural network. *Software Engineering*.

[2] Miao W. (2021). Emotional analysis of user online reviews based on text mining. *Scientific Journal of Economics and Management Research*.

[3] Yang S., Zhang N. (2021). Text sentiment analysis based on sentiment lexicon and context language model. *Journal of Computer Applications*.

[4] Sudhir P., Suresh V. D. (2021). Comparative study of various approaches, applications and classifiers for sentiment analysis. *Global Transitions Proceedings*.

[5] Pu Z. (2020). *E-Commerce Review Sentiment Analysis System Based on Machine learning*.

[6] Feng X. (2019). *Sentiment Analysis of Commodity Reviews Based on Deep Neural Network*.

[7] Dashtipour K., Gogate M., Adeel A., Larijani H., Hussain A. (2021). Sentiment analysis of Persian movie reviews using deep learning. *Entropy*.

[8] Kim H., Jeong Y.-S. (2019). Sentiment classification using convolutional neural networks. *Applied Sciences*.

[9] Alireza G., Karim S. M., Farzin Y. (2022). Ensemble transfer learning-based multimodal sentiment analysis using weighted convolutional neural networks. *Information Processing & Management*.

[10] Khan L., Amjad A., Afaq K. M., Chang H. T. (2022). Deep sentiment analysis using CNN-LSTM architecture of English and roman Urdu text shared in social media. *Applied Sciences*.

[11] Gao Z., Li Z., Luo J., Li X. (2022). Short text aspect-based sentiment analysis based on CNN + BiGRU. *Applied Sciences*.

[12] Kalaiarasu M., Kumar C. R. (2022). Sentiment analysis using improved novel convolutional neural network (SNCNN). *International Journal of Computers, Communications & Control*.

[13] Wang C., Liu Y. (2018). Research on emotional tendency of Chinese text based on doc2vec and deep neural network. *Electronic Technology & Software Engineering*.

[14] Fan S. (2021). *Design and Implementation of Intelligent Customer Service Quality Inspection System Based on Customer Sentiment Analysis*.

[15] Zheng C., Xie Z., Xing G., Chen S., Chen Y. (2021). Application of text classification technology in newspaper intelligent customer service system. *China Media Technology*.

[16] Cen Y., Li W., Li Li (2021). Research on multi-faceted emotion classification of e-commerce comment information based on deep learning. *Information Science*.

[17] Han L. (2021). *Research and Implementation of Sentiment Analysis Based on User Comments*.

[18] Guo X., Zhao N., Cui S. (2020). Consumer reviews sentiment analysis based on CNN-BiLSTM. *Systems Engineering-Theory & Practice*.

[19] Hu Y., Tong T., Zhang X., Peng J. (2022). Self-attention-based BGRU and CNN for sentiment analysis. *Computer Science*.

[20] Zhang M. Li S. (2021). Text sentiment classification model based on Bert-BiR-A neural network. *Video Engineering*.

[21] Luo S., Gu Y., Yao X., Fan W. (2021). Research on text sentiment analysis based on neural network and ensemble learning. *RIA*.

[22] Sun M., Li Y., Yu D., Zhang E., Li Q. (2019). Emotional analysis based on CNN-LSTM movie review. *Journal of Luoyang Institute of Science and Technology (Natural Science Edition)*.

[23] Wang L., Liu C., Cai D., Zhao T., Wang M. (2019). Text sentiment analysis based on CNN-BiLSTM network and attention model. *Journal of Wuhan Institute of Technology*.

[24] Yuan H., Zhang X. u., Niu W., Cui K. (2019). Sentiment analysis based on Multi-channel Convolution and Bi-directional GRU with attention mechanism. *Journal of Chinese Information Processing*.

[25] Chen N., Shan J., Wang J., Shi L. (2021). Sentiment analysis and research of user comments on hongmeng system based on text mining. *Science &Technology Information*.

[26] Miao Y., Ji Y., Peng E. Application of CNN-BiGRU Model in Chinese short text sentiment analysis.

[27] Xv Z., Yu Z., Yongfeng D (2020). Research on the construction of knowledge graph for film critic sentiment analysis. *Computer Simulation*.

